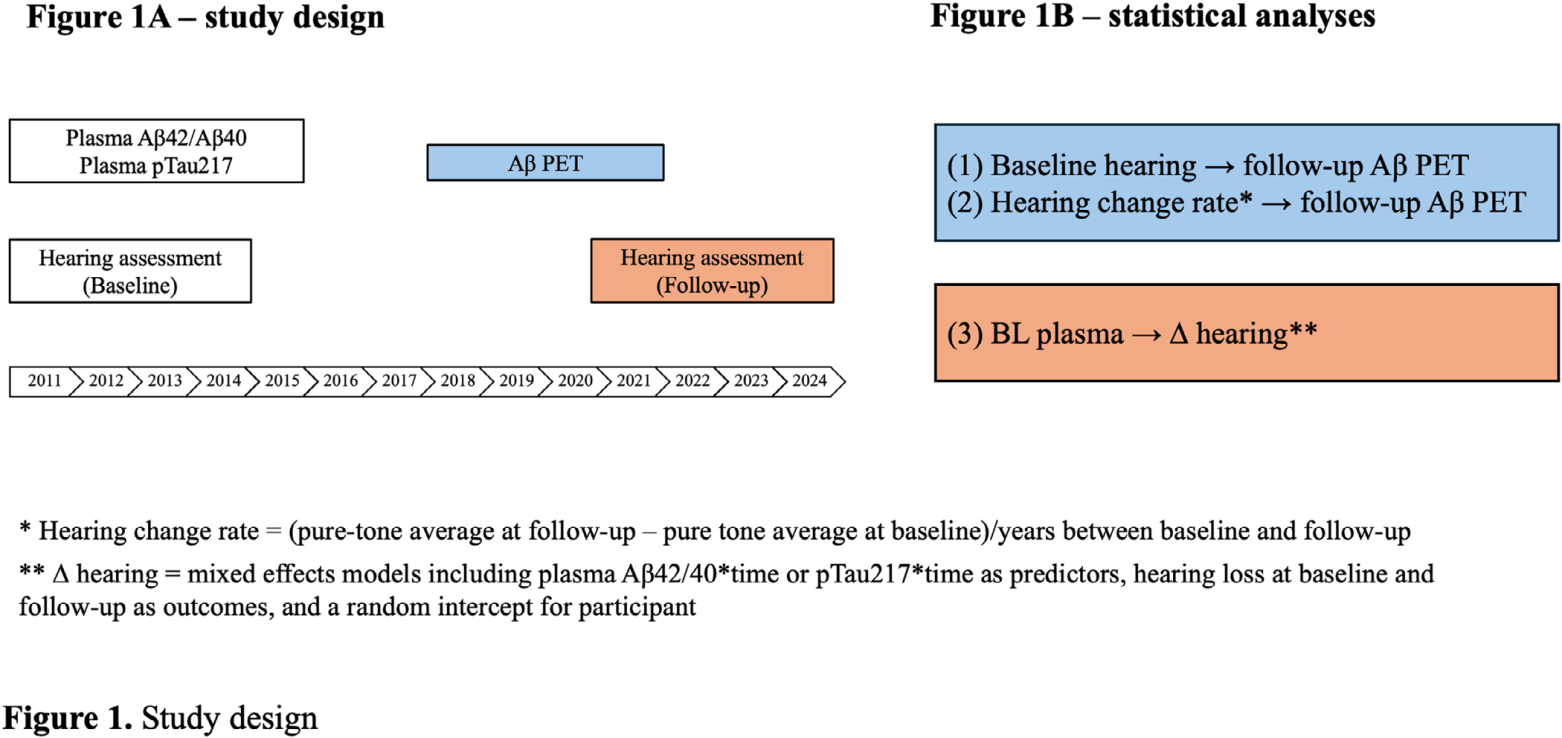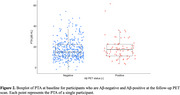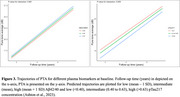# Hearing Loss and Amyloid Pathology: A Bidirectional Study

**DOI:** 10.1002/alz70860_103302

**Published:** 2025-12-23

**Authors:** Phuong Thuy Nguyen Ho, Jordi H.C. Boons, Arfan Ikram, Meike W. Vernooij, André Goedegebure, Julia Neitzel

**Affiliations:** ^1^ Erasmus MC, Rotterdam, Netherlands; ^2^ Erasmus University Medical Center, Rotterdam, Netherlands; ^3^ Harvard T.H. Chan School of Public Health, Boston, MA, USA

## Abstract

**Background:**

Age‐related hearing loss is a potential risk factor for dementia. While some studies have indicated a specific link to Alzheimer's disease, the relationship between hearing loss and amyloid‐β (Aβ) pathology remains inconclusive. This is further complicated by animal studies reporting a bidirectional relationship. The current study aimed to clarify the association between hearing loss and Aβ, and its directionality, in a human setting of community dwelling subjects.

**Method:**

At baseline, single molecule array plasma measures of Aβ42/40 and pTau217 were collected from 474 participants of the prospective population‐based Rotterdam Study (mean age = 62.37, 51.3% female). Participants underwent pure‐tone audiometry, using the pure‐tone average (PTA) of the better hearing ear as a measure for age‐related hearing loss. After on average seven years, these participants underwent amyloid 18F‐florbentaben PET. Two years after PET, PTA was measured a second time (Figure 1A). We investigated the association between (1) PTA at baseline and (2) changes in PTA with the risk of plasma‐negative participants (baseline pTau217<0.63 pg/mL, *n* = 461) converting to amyloid PET positivity during follow‐up (Figure 1B). We also studied the reverse relationship whether plasma biomarkers at baseline contribute to changes in PTA over time.

**Result:**

Baseline PTA was not a significant predictor of incident amyloid PET positivity seven years later (OR=0.96 [0.72, 1.28], *p* = 0.780). Similarly, yearly rate of change in PTA showed no association with incident amyloid PET positivity (OR=0.99 [0.72, 1.36], *p* = 0.952). Furthermore, no significant association was observed between baseline plasma biomarkers and change in PTA over time (Aβ42/40*time: β=0.0 [‐0.005, 0.006], *p* = 0.926; pTau217*time: β=0.001 [‐0.004, 0.007], *p* = 0.659.

**Conclusion:**

In this longitudinal cohort study, we found no (bidirectional) association between age‐related hearing loss and Aβ pathology. Our results suggest that hearing loss may increase dementia risk through other pathways than Aβ pathology.